# Induction of Fos protein expression in spinal cord neurons of tumour-bearing rats

**DOI:** 10.1038/sj.bjc.6690554

**Published:** 1999-07

**Authors:** S Kergozien, J-G Delcros, H Jouan, J-P Moulinoux

**Affiliations:** Groupe de Recherche en Thérapeutique Anticancéreuse, CNRS ESA 6027, Affiliée INSERM, Institut de Recherche Contre le Cancer (IRCC), Faculté de Médecine, 2 Avenue du Pr. L Bernard, CS 34317, 35043 Rennes Cedex, France

**Keywords:** prostatic carcinoma, Fos, spinal cord, rat, pain

## Abstract

The absence of discernible abnormal symptoms such as pain, often leading to delayed diagnosis in cancer patients, may be indicative of a dysregulation in sensory transmission between the tumour and the central nervous system. We explored expression of Fos protein in spinal cord neurons in rats, during the development of the MAT-LyLu prostatic adenocarcinoma grafted on the hind limb. The tumour triggered the densest Fos labelling in the L3–L5 lumbar segments, ipsilateral to the grafted limb. The labelling, detected at day 5, increased until day 10 and dropped off thereafter. The ventral horn (except lamina IX) was the most densely labelled region. Histological examination of the grafted limbs demonstrated that no inflammatory reaction accompanied the tumour growth. Rats exhibited no behavioural alterations either spontaneous or induced by handling. These results demonstrate that signals are sent to the central nervous system by the peripheral tumour. Considering both the behavioural and histological observations, it is unlikely that spinal activity reflects a painful state. The nature of these signals, inefficient to trigger the appropriate reaction of the organism against the tumour, remain to be determined with regard to the pharmacologically active compounds synthesized and released by the tumour cells. © 1999 Cancer Research Campaign


*British Journal of Cancer* (1999) **80**(10), 1512–1517
© 1999 Cancer Research Campaign
Article no. bjoc.1999.0554

### Induction of Fos protein expression in spinal cord neurons of tumour-bearing rats

**S Kergozien, J-G Delcros, H Jouan and J-P Moulinoux**

Groupe de Recherche en Thérapeutique Anticancéreuse, CNRS ESA 6027, Affiliée INSERM, Institut de Recherche Contre le Cancer (IRCC), Faculté de Médecine, 2 Avenue du Pr. L Bernard, CS 34317, 35043 Rennes Cedex, France

**Summary** The absence of discernible abnormal symptoms such as pain, often leading to delayed diagnosis in cancer patients, may be indicative of a dysregulation in sensory transmission between the tumour and the central nervous system. We explored expression of Fos protein in spinal cord neurons in rats, during the development of the MAT-LyLu prostatic adenocarcinoma grafted on the hind limb. The tumour triggered the densest Fos labelling in the L3–L5 lumbar segments, ipsilateral to the grafted limb. The labelling, detected at day 5, increased until day 10 and dropped off thereafter. The ventral horn (except lamina IX) was the most densely labelled region. Histological examination of the grafted limbs demonstrated that no inflammatory reaction accompanied the tumour growth. Rats exhibited no behavioural alterations either spontaneous or induced by handling. These results demonstrate that signals are sent to the central nervous system by the peripheral tumour. Considering both the behavioural and histological observations, it is unlikely that spinal activity reflects a painful state. The nature of these signals, inefficient to trigger the appropriate reaction of the organism against the tumour, remain to be determined with regard to the pharmacologically active compounds synthesized and released by the tumour cells.

**Keywords**: prostatic carcinoma; Fos; spinal cord; rat; pain

One of the vital functions of the nervous system is to provide information about the occurrence of injury or dysregulation likely to threaten the integrity of the organism. By its inherent aversive nature, the sensation of pain contributes normally to this function (Meyer et al, 1994). In the cancer patient, however, it is common to diagnose the presence of a tumoural process several months or years after onset and often after metastatic dissemination. The absence of discernible abnormal symptoms such as painful sensations is often the origin of such delayed diagnosis (Krakowski et al, 1996). Since cancer can be viewed as a breakdown in cellular communication processes (Morgan and Curran, 1991), the absence of such signs may be indicative of a dysregulation in the sensory transmission between the tumour and/or its environment and the central nervous system so that no signal would be provided to inform the organism about the existence of potential injury. Either the presence of a tumoural process does not provoke functional alterations in the nervous system activity, or the nervous system fails to respond appropriately to signals from the tumour, these failures resulting, at least during the first steps of development, in an insidious tumoural proliferation.

Owing to the total lack of experimental studies focusing on this topic, the main goal of the present study was to find out whether a tumour, the MAT-LyLu prostatic adenocarcinoma (Isaacs et al, 1981) grafted in the hind limb of rats, could be the origin of particular sensory information to the spinal cord, the first synaptic structure crossed by peripheral afferent inputs, using the expression of the c-*fos* gene as a marker of neuronal activity (Hunt et al, 1987; Morgan et al, 1987). The c-*fos* proto-oncogene, which becomes rapidly and transiently expressed following calcium influx through voltage-gated channels (Morgan and Curran, 1986), is closely linked to a messenger cascade coupling cell stimulation to gene expression (Morgan and Curran, 1991). Since numerous studies have emphasized the utility of monitoring Fos protein expression in spinal post-synaptic neurons to map the pattern of neuronal input to spinal sites (Hugues and Dragunow, 1995; Munglani and Hunt, 1995) Fos-like immunoreactivity (FLI) was evaluated in spinal cord neurons of tumour-bearing rats at various times of tumour development. Histological analyses were conducted in parallel to determine changes occurring during the tumour growth in the grafted hind limbs.

### MATERIALS AND METHODS

### Chemicals

Standard rodent chow was obtained from Pietrement (Provins, France), rabbit anti-Fos polyclonal IgG from Santa Cruz Biotech. (sc-52; Santa Cruz, CA, USA), avidin–biotin–peroxidase complex anti-rabbit IgG from Vector Laboratories (Burlingame, CA, USA). Other usual laboratory chemicals and reagents were from Sigma Chemical Co. (St Louis, MO, USA).

### Rats and tumour cells

All animal procedures were performed in compliance with strict institutional guidelines. Experiments were performed on 3-month-old male Copenhagen rats (200–250 g), housed at standardized conditions (20°C; alternating 12 h light–dark cycle) with chow

*Received 14 July 1998*
*Accepted 4 February 1999*

*Correspondence to:* J-G Delcros and fluid given ad libitum. MAT-LyLu prostatic adenocarcinoma cells were routinely maintained in culture at 37°C under 5% carbon dioxide atmosphere, in RPMI-1640 medium (Eurobio, Les-Ullis, France) supplemented with L-glutamine (2 mM), penicillin (100 U $ml^{-1}$), streptomycin (50 µg $ml^{-1}$) (Biomérieux, Marcy-l'étoile, France) and 10% heat-inactivated fetal calf serum. A tumoural process was induced by intramuscular inoculation of $2.10^6$ viable carcinoma cells in the right hind limb of the rat. Healthy control rats received an equal volume of saline vehicle. Tumour volume was determined with callipers as previously reported (Moulinoux et al, 1984). Rats were killed at various post-inoculation periods: 5, 10, 15 and 20 days after tumour graft.

### Histology

Tumours were systematically removed, immediately fixed in 10% formaldehyde and then embedded in paraffin. Four-micrometer sections obtained with a microtome were spread on glass slides and dried at 37°C during 48 h. Dewaxing was performed by immersion in toluene. After progressive rehydration (immersion in ethanol, then in phosphate-buffered saline (PBS)), slides were stained with haematoxylin-eosin and saffron, and then examined.

### Immunohistochemistry

Animals were deeply anaesthetized and perfused intracardially with 50 ml 0.1M PBS followed by 500 ml of 4% paraformaldehyde in 0.1 M phosphate buffer (PB) pH 7.4. The spinal cord was removed, post-fixed for 2 h in the same fixative, cryoprotected overnight in 30% sucrose in PB at 4°C, frozen in isopentane cooled down to –70°C with liquid nitrogen and kept at –80°C. Frozen 40-µm frontal sections were cut and collected in PB. The serial sections were immunostained for Fos-like protein using the avidin–biotin–peroxidase method (Hsu et al, 1981). The tissue sections were incubated for 30 min at room temperature in a blocking solution of 10% normal goat (NG) serum in PBS with 0.3% Triton-X (10% NGST); the sections were then incubated for 48 h at room temperature in the anti-Fos IgG diluted at 1:2000 in 1% NGST. After three washes in 1% NGST, sections were incubated overnight at 4°C with biotinylated goat anti-rabbit IgG diluted at 1:200 in 1% NGST, then washed twice in 1% NGST and incubated for 1 h with avidin–biotin–peroxidase complex. Finally, the sections were washed three times in PB, developed for 2 min in a solution of PBS containing 0.2 mg $ml^{-1}$ diaminobenzidine and 14 µl $ml^{-1}$ hydrogen peroxide, and washed three times in cold PB to stop the reaction. Sections were mounted on gelatine-subbed slides, dried at room temperature before being transferred in alcohol, then in xylene and finally coverslipped.

### Fos-labelled cell counts

Tissue sections were first examined using dark-field microscopy to determine the segmental level as previously described (Molander et al, 1984), and the grey matter landmarks. The sections were then examined under light-field microscopy to localize Fos-positive cells. Labelled nuclei were counted using a camera lucida attachment. The Fos-like immunoreactive neurons were observed from the lower thoracic to the upper sacral segments and studied more particularly through the L2–L5 lumbar spinal segments where most Fos-positive neurons were localized. For each rat, three sections per segment were examined. Referring to Presley et al (1990) the number of Fos immunoreactive neurons in the spinal grey matter was determined in the four regions defined: superficial dorsal horn (Laminae I and II), nucleus proprius (Laminae III and IV), neck of the dorsal horn (Laminae V and VI) and the ventral grey matter (Laminae VII, VIII, IX and X). Throughout the data collection phase, the investigator was blind to time of graft. Statistical analysis was made, using the non-parametric Mann–Whitney ranking test.

### RESULTS

### Growth rate of the tumour

Tumoural growth is reported in Table 1. The MAT-LyLu prostatic adenocarcinoma became palpable within 5 days and continued to grow rapidly until 15 days post-injection. The growth rate slowed down during the last survival 5 days.

### Histological observations

Histological data of grafted limbs at various time after tumour graft are reported in Table 2. Stained sections showed that all constitutive components of the limb were progressively infiltrated by tumour cells until their total destruction after 20 days of growth. The period ranging from 5 to 10 days post-inoculation, when tumour growth was exponential, was characterized by a centrifuge proliferation of tumour cells, with no or very moderate necrosis and bleeding. Important intra-tumoural haemorrhages and wide necrotic fields were observed at day 20. The intramuscular innervation remained unaffected until day 10. Twenty days after the graft, no nerves could be observed inside the tumoural tissues and nerves observed at the periphery were squashed. No major inflammatory reaction accompanied the tumour development. Little oedema (score 1 on a scale of 0–4) was observed at the periphery of the tumour till day 10 and was absent (score 0) thereafter. At every stage of the tumour growth, the density of inflammatory cells was very low inside the tumour except in necrotic areas where numerous polynuclear leucocytes were found.

### General behavioural observations

With the exception of cachexy which appeared after 2 weeks of tumour growth, rats did not manifest abnormal spontaneous behaviour. No vocalization and/or aggressiveness was spontaneously observed either during walking or when the tumour volume was measured with callipers, an experimental situation which needs a weak mechanical pressure over the grafted limb. No particular biting or licking of the grafted limb were observed even when the tumour volume has largely expanded. During

**Table 1** Growth of the MAT-LyLu adenocarcinoma grafted in rats (mean ± s.e.m.)

| Days after graft | Tumour size ($cm^3$) |
|---|---|
| 5 | 1.4 ± 0.3 |
| 10 | 3.4 ± 0.5 |
| 15 | 10.9 ± 2.3 |
| 20 | 18.0 ± 3.3 |

© 1999 Cancer Research Campaign

**Table 2** Histological modifications occurring in grafted limbs during the time course of MAT-LyLu growth

| | Time after graft | | |
|---|---|---|---|
| | 5 days | 10 days | 20 days |
| Tumoural tissue | Small well-individualized foci of proliferating cells; Centrifuge growth | Well-formed tumours Centrifuge growth | Major proliferation |
| Intra-tumoural necrosis | + | + | ++++ |
| Intra-tumoural bleeding | 0 | 0 | ++++ |
| Angiogenesis | ++ (tumoural, peripheral) | ++ (intra-tumoural) | ++++ (intra-tumoural) |
| Peripheral tissue (muscle) | Invasion by tumour cells with little muscle destruction | Considerable centrifuge muscle destruction | Complete destruction of muscle tissue |
| Innervation (muscle) | Normal | Normal | Total destruction inside the tumour; peripheral nerves compressed |

locomotion both hind paws were on the floor, at least until the third week when the size of the tumour became an indrance for a normal use of the limb.

### Fos expression in tumour-bearing rats

Growth of the MAT-LyLu carcinoma in the hind limb induced the expression of the Fos protein in spinal grey matter. Whereas in healthy rats the few labelled neurons detected were sparsely and bilaterally distributed throughout the entire grey matter, in tumour-bearing rats Fos-immunoreactive neurons were mainly confined to the side of the spinal cord ipsilateral to the grafted hind limb and more particularly in the lumbar segments (Figures 1 and 2). Rostral and caudal to the lumbar enlargements the number of positive cells rapidly diminished and no difference in the Fos-like immunoreactivity was detected between healthy and tumour-bearing rats at these levels (data not shown). Fos-like immunoreactivity (FLI) evoked by tumour graft was time dependent (Figure 2). By 5 days after graft, while tumours were still scarcely palpable (Table 1), the number of Fos-immunoreactive neurons increased twofold in the ipsilateral side as compared with healthy rats. Maximum Fos-like immunoreactivity was observed 10 days after tumour cell inoculation, both in terms of numbers of positive neurons (threefold increase), and in labelling intensity. Thereafter FLI dropped although the tumour was still expanding. The decrease observed 15 days after tumour graft was more marked 5 days later: after 20 days of tumour growth, Fos labelling dropped by 50% and tended to control values (Figure 2).

Analysis of the rostrocaudal distribution of FLI showed that from 5 to 15 days of tumour growth, most labelled neurons were located between the L3 and the L5 spinal segment levels with a maximum in the L4 segment (Figure 3). Twenty days after tumour graft, a similar number of Fos-immunoreactive neurons were observed in the four lumbar segments.

Laminar distribution of Fos labelling in segment L4, showed major differences depending on the time course of tumour

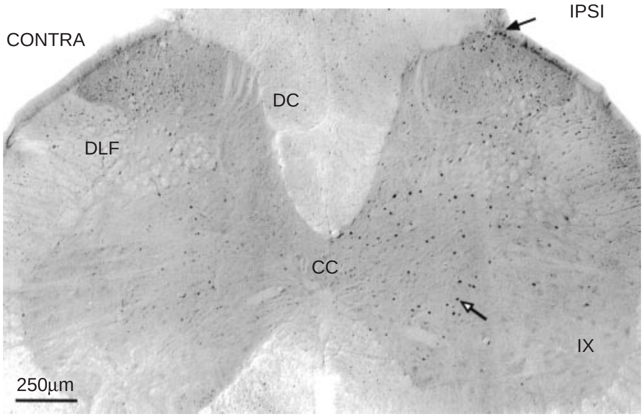

**Figure 1** Photomicrograph illustrating the location of Fos-like immunoreactive neurons, in a 40 µm section of L4 segment, in tumour-bearing rat 10 days after graft. Note that the densest labelling is present on the side of the spinal cord ipsilateral to the grafted hindlimb. Black arrow points to labelled cells in the superficial dorsal horn; white arrow points to labelled cells in the ventral horn. CC, central canal; DC, Dorsal columns; DLF, Dorsolateral funiculus

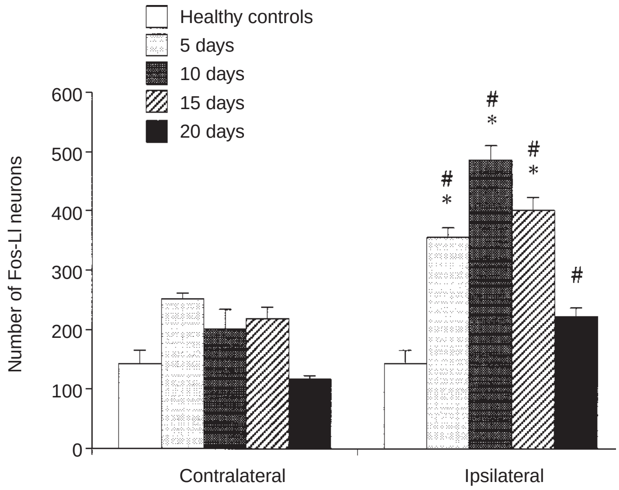

**Figure 2** Histogram presenting the total number of Fos immunoreactive neurons in the lumbar spinal enlargement, from L2 to L5 in the two sides of the spinal cord, at different stages of the tumour growth. Five groups of rats are considered: control rats ($n$ = 4) and tumour-bearing rats, 5 days ($n$ = 4), 10 days ($n$ = 4), 15 days ($n$ = 4) and 20 days ($n$ = 4) after graft. Results are expressed as mean (± s.e.m.) number of Fos immunoreactive neurons for four lumbar segments per 3 × 40-µm sections. Significance is expressed taking as reference the control group (*$P$ < 0.05) or the respective contralateral side (#$P$ < 0.05)

© 1999 Cancer Research Campaign

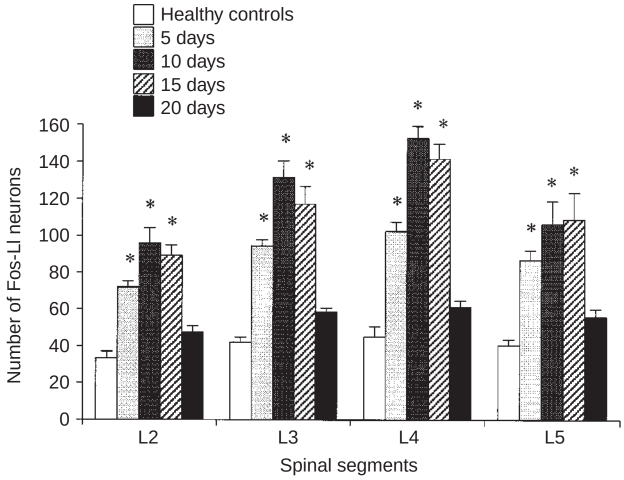

**Figure 3** Histogram presenting the rostrocaudal distribution in the lumbar enlargement (from L2 to L5) of the number of Fos immunoreactive neurons at different times after tumour graft. Results are expressed as mean (± s.e.m.) number of Fos immunoreactive neurons for four lumbar segments per 3 × 40-µm sections. Significance is expressed taking as reference the control group (**P* < 0.05)

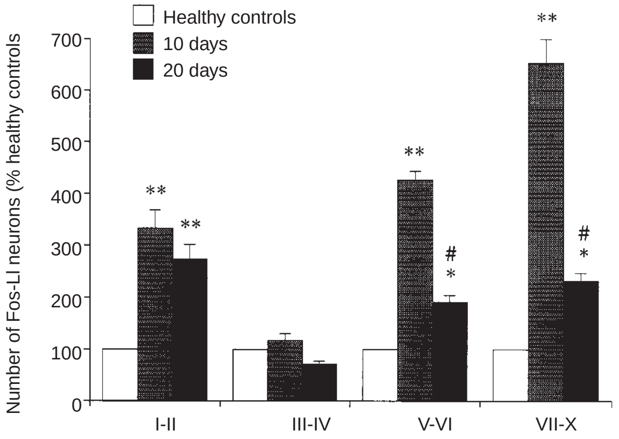

**Figure 4** Effects of the tumour graft on the laminar distribution of the Fos immunoreactive neurons in the ipsilateral L4 segment. Comparison between control (*n* = 6) and tumour-bearing rats, 10 days (*n* = 6) and 20 days (*n* = 7) after graft. Results are expressed as mean (± s.e.m.) percentages of control values of Fos immunoreactive neurons. Significance is expressed taking as reference the control group (**P* < 0.05; ***P* < 0.01) or tumour-bearing rats after 10 days of graft (# *P* < 0.01)

development (Figure 4). By 10 days of tumour growth, a threefold increase in the number of Fos-immunoreactive neurons was observed in the superficial dorsal horn (laminae I and II). Labelled neurons were sparsely distributed into these laminae and were not concentrated in any particular area. A high density of labelled neurons was also found in the neck of the dorsal horn (laminae V and VI) where their number was enhanced fourfold. The most extensive Fos labelling was nevertheless observed in the ventral grey matter (sixfold increase) and more particularly in laminae VII, VIII and around the central canal (X). Lamina IX was devoid of labelling. In the nucleus proprius (laminae III and IV), no significant changes were found between healthy and tumour-bearing rats. After 20 days of graft, the Fos-like immunoreactivity dropped significantly in deep laminae, i.e. in the neck of the dorsal horn and in ventral regions of the spinal cord. In contrast, no significant drop of FLI was observed between 10 and 20 days of graft in the superficial dorsal horn.

### DISCUSSION

Our present results demonstrate that a tumour growing in the hind limb of rat triggers spontaneously, that is in absence of any deliberate additional stimulation, the expression of the Fos protein in specific regions of the spinal cord. Most labelled neurons evoked by the tumour graft were detected ipsilaterally at the L3–L5 levels, consistent with the somatotopic organization of the primary afferent fibres that innervate the grafted hind limb (Molander et al, 1984; Swett and Woolf, 1985). Because spinal Fos expression reflects neuronal activity (Hunt et al, 1987; Morgan et al, 1987), the present observations demonstrate the existence of a primary afferent transmission from the peripheral tumour and/or the adjacent tissues to the spinal cord. The persistence of Fos expression for at least 2 weeks after tumour graft, that is well beyond the half-life of the Fos protein (Müller et al, 1984) is indicative of continuous afferent drive and/or of the development of spontaneous/central activity of neurons in the spinal cord. Fos-like immunoreactivity evoked by the tumour graft was time-dependent. As soon as 5 days after graft, when tumours were still scarcely palpable, a significant number of labelled neurons were detected, demonstrating that sensory information is transmitted at an early stage of tumour growth. Maximum Fos labelling was observed 10 days after graft when the tumour had the highest rate of growth. Thereafter, the tumour was still expanding, though more slowly, and the Fos labelling dropped. This bell-shaped time-course of Fos expression may be indicative of modifications in signal transmission and/or in central activity. The nature of these signals sent by the tumour and which provoked functional alterations in the spinal cord activity remains then to be determined.

Since the first report by Hunt et al (1987), spinal Fos expression has been largely used as a neuronal marker of pain (Harris, 1998). However, non-noxious stimulation also triggers spinal Fos-like immunoreactivity (Jasmin et al, 1994). Fos expression should be considered as a general marker of neuronal activity rather than a specific marker of pain. Regarding the general behaviour, the tumour-bearing rats did not manifest any discernible signs of pain either spontaneously or during manipulation whatever the stage of tumour growth. Thus, rats did not manifest particular behaviour compared to control when the tumour size was evaluated. Consistent with these observations, our histological studies of the grafted limbs revealed that no inflammatory reaction accompanied the tumour growth. From the foregoing observations it seems then unlikely that spinal Fos expression observed in tumour-bearing rats is related to pain. Besides, the general pattern of Fos immunoreactivity in the spinal grey matter of grafted rats differed in several aspects from those observed after chronic injury-induced pain. After 5–10 days of graft, when the tumour rapidly expanded, Fos labelling was detected in the superficial laminae, in the neck of the dorsal horn, but the maximum immunoreactivity was located in deeper laminae (VII, VIII) and around the central canal (X). These findings contrast with neuropathic models of pain where Fos expression predominates in the nucleus proprius (laminae III and IV) (Molander et al, 1992; Chi et al, 1993). Conversely, in models of chronic inflammatory pain, such as polyarthritis, labelled neurons are mainly located in the neck of the dorsal horn (Abbadie and Besson, 1992). The observed localization of Fos-like immunoreactive neurons in tumour-bearing rats

© 1999 Cancer Research Campaign would best fit the pattern observed in monoarthritic rats (Lanteri-Minet et al, 1993) even if the time-courses differ. In tumour-bearing rats, the detection of Fos labelling in nociceptive responding areas (Besson and Chaouch, 1987) is not in conflict with our previous suggestions since these areas are not specific for pain (Bennet et al, 1980; Menetrey, 1987). Thus, the presence of a tumoural process provokes functional alterations in spinal cord activity, demonstrating that a tumour actively proliferating in the limb sends afferent signals to the central nervous system, signals which are probably unrelated to pain.

The tumour context is a quite complex situation with multiple components which may have an impact on central nervous system activity. Mechanical compression of the neighbouring muscles and nerves as well as overstretching of the surrounding skin may constitute important stimuli. Nevertheless, spinal Fos was intensely expressed at an early stage of tumour growth, when these mechanical factors are minimal. Pharmacologically active compounds synthesized and released by the tumour cells are probably involved in this neuronal activity. In particular, polyamines, biogenic amines with important regulatory functions in the proliferation of malignant cells (Jänne et al, 1978; Marton and Pegg, 1995), are also known to play physiological roles in intracellular signal processing and neurotransmission. Among the polyamines, spermine and to a lesser extent spermidine affect neuronal excitability by interacting with ion channels and/or receptors coupled to these channels, including some glutamate receptors (NMDA, AMPA/Kainate) that drive neurons (reviewed in Williams, 1997). Recent reports have evidenced the presence of these receptors on peripheral sensory afferents (Carlton et al, 1995; Zhou et al, 1996). E-type prostaglandins have also been detected in tumour effusion of the MAT-LyLu adenocarcinoma (Clayton et al, 1985; Shaw et al, 1985). These compounds interact on several types of ion channels to sensitize sensory neurons (Martin et al, 1987). Despite the release of these well-known inflammatory compounds, the growth of the MAT-LyLu adenocarcinoma is not accompanied by an inflammatory reaction. Because spermine has recently been shown to protect mice against the development of carrageenan-induced inflammation (Zhang et al, 1997), it is tempting to speculate that the facilitatory effect of E-type prostaglandins may be counterregulated by polyamines present in great amount in the tumour. Taken together, these observations may suggest that peripheral afferent fibres would be less sensitive to modifications of their environment or would convey altered peripheral information. Spinal neuronal activity in these conditions would reflect these modified afferent inputs. Such a hypothesis could explain the similar topology of spinal activity observed in tumour-bearing and monoarthritic rats. The tumour development would also drive the polymodal c-fibres. And nevertheless, the information did not seem to be perceived as such since tumour-bearing rat did not exhibit any characteristics associated with inflammation.

In conclusion, these results demonstrate that signals are sent to the central nervous system by the peripheral MAT-LyLu tumour. But, considering both the behavioural and histological observations, it is unlikely that spinal activity reflects a painful state. Actually we cannot rule out the possibility of a central effect. However, it is tempting to speculate that peripheral tumours may send altered signals to the central nervous system, masking in some way the existence of tumour-induced injuries. The nature of these signals, inefficient to trigger the appropriate reaction of the organism against the tumour, remain to be determined with regard to the pharmacologically active compounds synthesized and released by the tumour cells.

### ACKNOWLEDGEMENTS

This work was supported by grants from CNRS and the Ligue Nationale Contre le Cancer (Comité d'Ille-et-Vilaine). S Kergozien was a recipient of a grant from the Ligue Nationale Contre le Cancer (Comité des Côtes d'Armor). The authors are indebted to Dr D Menetrey for helpful discussions, R Rambur for the photomicrograph and gratefully acknowledge Dr M Bureau and Dr L Chamaillard for comments on the manuscript.

### REFERENCES

Abbadie C and Besson J-M (1992) c-*fos* expression in rat lumbar spinal cord during the development of adjuvant-induced arthritis. *Neuroscience* **48**: 985–993

Bennet GJ, Abdelmoumene M, Hayashi H and Dubner R (1980) Physiology and morphology of substantia gelatinosa neurons intracellularly stained with horseradish peroxidase. *J Comp Neurol* **194**: 809–827

Besson J-M and Chaouch A (1987) Peripheral and spinal mechanisms of nociception. *Physiol Rev* **67**: 67–186

Carlton SM, Hargett GL and Coggeshall RE (1995) Localization and activation of glutamate receptors in unmyelinated axons of rat glabrous skin. *Neurosci Lett* **197**: 25–28

Chi SI, Levine JD and Basbaum AI (1993) Peripheral and central contributions to the persistent expression of spinal cord Fos-like immunoreactivity produced by sciatic nerve transection in the rat. *Brain Res* **617**: 225–237

Clayton M, Rubenstein M, Shaw M and Guinan P (1985) Prostaglandin $E_2$ activity in the microenvironment of prostate tumours. *Surg Forum Urol* **36**: 644–645

Harris JA (1998) Using *c-fos* as a neural marker of pain. *Brain Res Bull* **45**: 1–8

Hsu S, Raine L and Fanger H (1981) A comparative study of the peroxidase–antiperoxidase method and an avidin–biotin complex method for studying polypeptide hormones with radioimmunoassay antibodies. *Am J Clin Pathol* **75**: 734–738

Hugues P and Dragunow M (1995) Induction of immediately-early genes and the control of neurotransmitter-regulated gene expression within the nervous system. *Pharmacol Rev* **47**: 133–178

Hunt SP, Pini A and Evan G (1987) Induction of c-Fos-like protein in spinal cord neurons following sensory stimulation. *Nature* **328**: 632–634

Isaacs JT, Yu GW and Coffey DS (1981) The characterization of a newly identified, highly metastatic variety of dunning R 3327 rat prostatic adenocarcinoma system: the MAT-LyLu tumour. *Invest Urol* **19**: 20–23

Jänne J, Poso H and Raina A (1978) Polyamines in rapid growth and cancer. *Biochim Biophys Acta* **473**: 241–293

Krakowski I, Gestin Y, Jaulmes F, Lakadja F, Meynadier J, Poulain P, Borgo CPD, Rebattu P, Schach R, Goldberg J, Boureau F, Falcoff H, Guillain H, Larue F, Magnet M, Salamagne M, Serrie A, Trechot P and Verdie JC (1996) Recommandations pour une bonne pratique dans la prise en charge de la douleur du cancer chez l'adulate et l'enfant. *Bull Cancer* **83**: (Suppl. 1).

Lanteri-Minet M, De Pommery J, Herdegen T, Weil-fugazza J, Bravo R and Menetrey D (1993) Differential time course and spatial expression of Fos, Jun and Krox-24 proteins in spinal cord of rats undergoing subacute or chronic somatic inflammation. *J Comp Neurol* **333**: 223–235

Martin HA, Basbaum AI, Kwiat GC, Goetzl EJ and Levine JD (1987) Leukotriene and prostaglandin sensitization of cutaneous high-threshold C- and A-delta mechanonociceptors in the hairy skin of rat hindlimbs. *Neuroscience* **22**: 651–659

Marton LJ and Pegg AE (1995) Polyamines as targets for therapeutic intervention. *Annu Rev Pharmacol Toxicol* **35**: 55–91

Menetrey D (1987) Spinal nociceptive neurons at the origin of long ascending pathways in the rat: electrological, anatomical and immunohistochemical approaches. In: *Thalamus and Pain*, Besson J-M, Guibault G and Peschanski M (eds), pp. 21–34. Excerpta Medica: Amsterdam.

Meyer RA, Campbell JN and Raja SN (1994) Peripheral neural mechanisms of nociception. In: *Textbook of Pain*, Wall PD and Melzack R (eds), pp. 13–44. Churchill Livingstone: Edinburgh.

Molander C, Xu Q and Grant G (1984) The cytoarchitectonic organization of the spinal cord in the rat. I. The lower thoracic and lumbosacral cord. *J. Comp. Neurol* **230**: 133–141

 © 1999 Cancer Research Campaign

Molander C, Hongpaisan J and Grant G (1992) Changing pattern of *c-fos* expression in spinal cord neurons after electrical stimulation of the chronically injured sciatic nerve in the rat. *Neuroscience* **50**: 223–236

Morgan JI and Curran T (1986) Role of the ion flux in the control of *c-fos* expression. *Nature* **322**: 552–555

Morgan JI and Curran T (1991) Stimulus-transcription coupling in the nervous system: involvement of the inducible proto-oncogenes *fos* and *jun*. *Ann Rev Neurosci* **14**: 421–451

Morgan JI, Cohen DR, Hempstead JL and Curran T (1987) Mapping patterns of *cfos* expression in the central nervous system after seizure. *Science* **237**: 192–197

Moulinoux J-P, Quemener V, Larzul JJ, Calve ML, Roch AM, Toujas L and Quash JA (1984) RBC polyamines in mice bearing the Lewis lung carcinoma and in patients with bronchopulmonary cancers. *Int J Cancer* **34**: 277–281

Müller R, Bravo R, Bruckhardt J and Currant T (1984) Induction of *c-fos* gene and protein by growth factor precedes activation of *c-myc*. *Nature* **312**: 716–720

Munglani R and Hunt SP (1995) Proto-oncogenes: basic concepts and stimulation induced changes in the spinal cord. In: *Progress in Brain Research*, Nyberg F, Sharma HS and Wiesenfield-Hallin Z (eds), pp. 283–298. Elsevier: Amsterdam.

Presley RW, Menetrey D, Levine JD and Basbaum AI (1990) Systemic morphine suppresses noxious stimulus-evoked fos protein-like immunoreactivity in the rat spinal cord. *J Neurosci* **10**: 323–335

Shaw MW, Ablin ARJ, Ray P, Rubenstein M, Guinan PD and Mckiel CF (1985) Immunobiology of the dunning R-3327 rat prostate adenocarcinoma sublines: plasma and tumor effusion prostaglandins. *Am J Reprod Immunol Microbiol* **8**: 77–79

Swett JE and Woolf CJ (1985) The somatotropic organization of primary afferent terminals in the superficial laminae of the dorsal horn of horn of the rat spinal cord. *J Comp Neurol* **231**: 66–77

Williams K (1997) Interaction of polyamines with ion channels. *Biochem J* **325**: 289–297

Zhang M, Caragine T, Wang H, Cohen PS, Botchkina G, Soda K, Bianchi M Ulrich P, Cerami A, Sherry B and Tracey KJ (1997) Spermine inhibits proinflammatory cytokine synthesis in human mononuclear cells: a counterregulatory mechanism that restrains the immune response. *J Exp Med* **185**: 1759–1768

Zhou S, Bonasera L and Carlton SM (1996) Peripheral administration of NMDA, AMPA or KA results in pain behaviors in rats. *Neuroreport* **7**: 895–900

*© 1999 Cancer Research Campaign* 
